# Comparative safety and effectiveness of ultrasound-guided radiofrequency ablation combined with preoperative three-dimensional reconstruction versus surgical resection for solitary hepatocellular carcinoma of 3-5 cm

**DOI:** 10.7150/jca.32342

**Published:** 2019-09-03

**Authors:** Yuanfeng Gong, Yunqiang Tang, Yan Geng, Yu Zhou, Min Yu, Bowen Huang, Zhonghai Sun, Hui Tang, Zhixiang Jian, Baohua Hou

**Affiliations:** 1 Department of General Surgery, Guangdong Provincial People's Hospital, Guangdong Academy of Medical Sciences, No.106 Zhongshan 2nd Rd, Yuexiu Dist, Guangzhou 510080, China;; 2 Department of Hepatobiliary Surgery, the Affiliated Cancer Hospital & Institute of Guangzhou Medical University, No.78 Hengzhigang Rd, Yuexiu Dist, Guangzhou 510095, China;; 3 Department of Gastrointestinal Surgery, Shunde Hospital of Southern Medical University, No.1 Jiazi Rd, Shunde Dist, Foshan 528300, China.

**Keywords:** hepatocellular carcinoma, radiofrequency ablation, three-dimensional reconstruction, three-dimensional image fusion, hepatectomy

## Abstract

Objective: To investigate the safety and effectiveness of ultrasound-guided radiofrequency ablation (RFA) combined with preoperative three-dimensional (3D) reconstruction versus surgical resection for solitary hepatocellular carcinoma of 3-5 cm.

Methods: The cohort of this retrospective study included 66 consecutive patients who underwent open hepatectomy (Surgery group) between January 2009 and December 2014, as well as 54 consecutive patients who underwent ultrasound-guided RFA combined with preoperative 3D reconstruction (RFA group) during the same period. Preoperative 3D reconstruction was performed using Myrian-XP-Liver software. The image fusion system was used to evaluate the RFA safety margin at 1 month after surgery. Kaplan-Meier analysis and the log-rank test were used to compare the recurrence and overall survival (OS) rates between the two treatment groups.

Results: There were no significant differences in the baseline characteristics of the two groups. The complete ablation rate was 94.4% (51/53). As compared with surgical resection for solitary HCC of 3-5 cm, ultrasound-guided RFA combined with preoperative 3D reconstruction significantly reduced the morbidity of excessive pain, total complications, and infections (*p* < 0.001). A significant decrease in the duration of the hospital stay after treatment was also observed in the RFA group (*t* = 10.017, *p* < 0.001). There was no significant difference in the cumulative recurrence rate between the two groups. Kaplan-Meier analysis and the log-rank test revealed no significant difference in the OS rate between the two groups over a 3-year follow-up period.

Conclusion: Ultrasound-guided RFA combined with preoperative 3D reconstruction appears to be a safe and effective therapeutic option for patients with solitary HCC of 3-5 cm.

## Introduction

Hepatocellular carcinoma (HCC) is the sixth most common cancer and the second most common cause of cancer-related death worldwide, accounting for an estimated 782,500 new cases and 745,500 deaths annually 1. Although liver resection is the primary treatment for HCC, extensive surgical resection has certain limitations due to the risk of liver dysfunction after ischemia-reperfusion injury, the inflammatory response, nutrient metabolism abnormalities, and differences in liver cirrhosis status and resection range 2. In recent years, ultrasound-guided radiofrequency ablation (RFA) of small HCC has been widely applied in clinical practice. For lesions smaller than 3 cm, RFA has the same safety and effectiveness as surgical resection 3-4, while for lesions with a diameter of 3-5 cm, the curative effect remains controversial 5. Incomplete ablation is the main factor that affects its curative effect. Accurate preoperative imaging assessment can not only reduce the complication rate of RFA, but also ensure a tumor-free margin and improve survival 6. Preoperative three-dimensional (3D) reconstruction is used to stereoscopically display the tumor location, intrahepatic vasculature, and adjacent organs to better plan and improve the safety and effectiveness of RFA 7-8. Thus, the aim of this retrospective study was to comprehensively investigate the safety and effectiveness of ultrasound-guided RFA combined with preoperative 3D reconstruction versus surgical resection for HCC of 3-5 cm.

## Materials and Methods

### Subjects

The cohort of this retrospective study included 66 consecutive patients who underwent open hepatectomy between January 2009 and December 2014, as well as 54 consecutive patients who underwent ultrasound-guided RFA combined with preoperative 3D reconstruction during the same period. The study protocol was approved by the Ethics Committee of the Hospital and written informed consent was obtained directly from all participants. All subjects met the following criteria: (1) hepatectomy or RFA as the first-line treatment, but no previous chemotherapy or radiotherapy; (2) confirmed diagnosis of HCC by history, AFP level, image study, and postoperative pathological analysis; and (3) Child-Pugh grade A or B, with one tumor with a diameter of 3-5 cm, with no macrovascular invasion or distant metastasis. Exclusion criteria were as follows: (1) unavailability of pre- and post-operative computed tomography (CT) data, as some patients underwent preoperative enhanced CT in other hospitals and we could not obtain the digital CT data. (2) loss to follow-up within 3 months after RFA or surgery, (3) any other malignancy, or (4) the anatomical structure around the ablation area was obviously changed (e.g., massive ascites), which might affect the fusion of images.

### 3D reconstruction and RFA procedure

Preoperative enhanced CT was performed using the GE Lightspeed 64-slice helical CT system (GE Healthcare Life Sciences, Chicago, IL, USA). Myrian-XP-Liver software (Intrasense SA, Montpellier, France) was used for 3D reconstruction. Ultrasound-guided (Hitachi Aloka SSD-Alpha 5 Surgical Ultrasound System; Hitachi Ltd., Tokyo, Japan) percutaneous RFA was conducted with the Cool-tip RFA System (Medtronic plc, Dublin, Ireland) with a 17G electrode needle. Percutaneous liver biopsy was performed using a 16G Tru-Core needle (15 cm; Angiotech, Wheeling, IL, USA).

For CT assessment, all patients were placed in the supine position. The scanning area ranged from the diaphragm to the kidney. The scanning parameters were as follow: current, 250 mA; voltage, 120 kV; slice thickness, 5 mm; pitch, 0.984; and rotation speed, 0.5 s/cycle. The contrast agent was highly concentrated Ultravist 370 (Bayer Pharma AG, Wuppertal, Germany). The scanning was delayed 20-25 s after the arterial phase and 55-60 s after the venous phase. CT data of the arterial and venous phases, as well as the portal vein phase were transferred to the GE AW4.4 workstation, copied onto a DVD (DICOM format), and subsequently imported into Myrian-Xp-Live software. The system automatically identified and extracted image information of the liver parenchyma, tumors, hepatic artery, portal vein, and hepatic vein, and then generated reconstructed 3D images of the liver. The tumor location and size, as well as the adjacent vasculature could be clearly observed in all directions. Sometimes, the automatic identification was insufficient or excessive, thus appropriate cutting was manually performed. The liver volume was estimated with Myrian-Xp-Live software.

RFA procedure: The 3D RFA target area (a tumor-free margin of at least 5 mm) was designed according the preoperative 3D reconstructed image. It was not possible to reach such a safety margin in some cases owing to the adjacent vasculature, intestines, or gallbladder. Under local or general anesthesia, ultrasound-guided percutaneous liver biopsy was performed. Then, a 17G Cool-tip electrode needle was punctured into the liver under real-time ultrasound guidance. The radiofrequency power was gradually increased and lasted about 10 min. The puncture track was ablated once the procedure was finished in order to reduce postoperative bleeding and seeding of tumor cells. Sometimes, the electrode needle could not be accurately inserted when the tumor was located near the hepatic dome due to lower chest interference. For such cases, the artificial hydrothorax technique was applied by injecting 800-1000 mL of saline into the right thoracic cavity to push the diaphragm downwards. After the RFA procedure, Doppler ultrasound was used to detect the target area and determine whether the blood flow signal had still existed.

### Effect evaluation

Definitions of postoperative complications were as follows: Post-hepatectomy hemorrhage was defined as a decrease in postoperative hemoglobin levels of >3 g/dL, as compared with the baseline level and/or any postoperative transfusion of packed red blood cells and/or the need for radiological intervention (such as embolization), and/or re-operation to stop the bleeding. Evidence of intra-abdominal bleeding was obtained by imaging or blood loss via the abdominal drains, if present. Biliary leakage was defined as a drainage bilirubin to serum bilirubin ratio of > 3 at day 3 or after resection, or interventional surgical revision due to biliary peritonitis. Post-hepatectomy liver failure was defined as an increase in the international normalized ratio and hyperbilirubinemia on or after day 5. A clinical diagnosis of sepsis was defined as systemic inflammatory response syndrome with a documented infection. Systemic inflammatory response syndrome was demonstrated by two or more of the following four variables: (1) hyperthermia (> 38.3°C) or hypothermia (< 36°C); (2) tachycardia (> 90 beats/min); (3) tachypnea (rate ≥ 20 breaths/min) or hypoxia (oxygen saturation < 90% or need for oxygen supplementation of ≥ 0.4 fraction of inspired oxygen to maintain adequate saturation); and (4) leukocytosis [white blood cell (WBC) count of > 12.0 × 10^9^ cells/L], leukopenia (WBC count of < 4 × 10^9^ cells/L), or immature or band forms of > 10%. Postoperative ileus was defined as two or more of the following symptoms: nausea/vomiting, inability to tolerate an oral diet for more than 24 h, absence of flatus for more than 24 h, distension, and radiological confirmation on or after day 4 without prior resolution of ileus.

An increase in the WBC count was induced by the inflammatory reaction. Pre- and post-operative liver function was assessed using the Child-Pugh scoring system based on serum levels of alanine aminotransferase (ALT), aspartate transaminase (AST), total bilirubin (TBil), and albumin (Alb), as well as the prothrombin time (PT). Preoperative reserved liver function was measured according to the indocyanine green retention rate at 15 min (ICG15). Venous blood samples were obtained from all subjects before surgery and on postoperative days 1 (D1), 3 (D3), and 7 (D7). The preoperative characteristics of all patients [i.e., age, sex, body mass index, fasting blood glucose, hepatitis B surface antigen (HBsAg) status, Child-Pugh score, ICG15, and alpha-fetoprotein (AFP)], intraoperative characteristics (surgical duration, complete ablation rate, blood loss, and Pringle time), and postoperative characteristics (excessive pain, duration of postoperative hospital stay, complications, and mortality) were collected.

**Figure 1:**
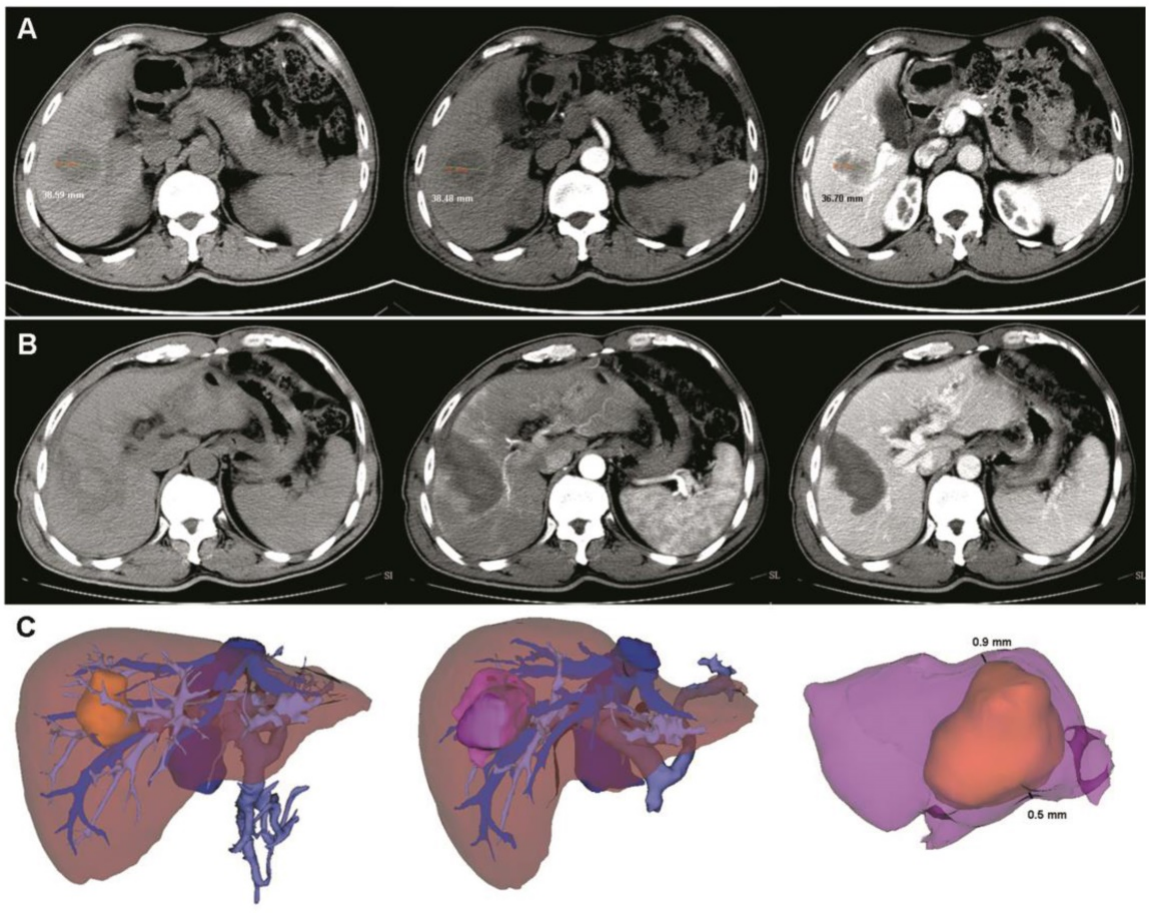
** Pre- and post- operative 3D-CT reconstruction and image fusion. (A)** Pre-operative two-dimension computed tomography (plain scan, arterial and venous phases). The tumor size was about 38.59 mm. **(B)** After ultrasound-guided RFA combined with 3D-CT reconstruction, a low-density area was observed (plain scan, arterial and venous phases). **(C)** Pre- and post-operative 3D-CT reconstruction and image fusion. The yellow mass indicates the tumor and the purple mass indicates the ablation area. After image fusion, the safety margin was measured in a multidimensional approach. In this case, the thinnest safety margin was 0.6 mm. RFA, radiofrequency ablation.

An image fusion system (Myrian-Xp-Live software), as described in our previous study 9, was used to evaluate the RFA safety margin at 1 month after surgery. Images of the ablation area and tumor were superimposed in a 3D model (**Figure 1**). The transparency of two models (per- and post-RFA) were adjusted and rotated to determine and measure the shortest tumor-free margin. A safety margin of 5 mm or more was considered as complete ablation, while less than 5 mm was considered as incomplete ablation 9.

### Follow-up

Tumor recurrence was diagnosed by enhanced CT and/or magnetic resonance imaging (MRI). Liver biopsies of new lesions were unnecessary. CT and/or MRI were performed at 4 weeks after treatment and every 2 month thereafter during the first 2 years. At each of these follow-up visits, blood tests including liver function tests and serum AFP were done. Chest radiography was done every 6 months. The follow-up visits were spaced out to once every 3 months after 2 years. Tumor recurrence was defined as a new enhanced mass in the arterial phase with washout in the portal site or a delayed phase. A mass showing arterial enhancement without washout was considered as a progressively growing mass on follow-up images or lipiodol retention after transcatheter arterial chemoembolization (TACE) as recurrent HCC. TACE or RFA was performed in the case of neoplasm recurrence. The recurrence time was calculated from the end of surgery to the diagnosis of tumor recurrence. The overall survival (OS) rate was calculated from the end of surgery to the time of death or the latest follow-up. The postoperative 1-, 2-, and 3-year progression-free survival (PFS) and OS rates were compared between the RFA and surgery groups.

### Statistical Analysis

Continuous variables are presented as the mean and standard deviation. The Student's *t*-test was used to identify differences between the means of two continuous variables. The *χ2* test or Fisher's exact probability test was used to determine whether the frequencies between the RFA and surgery groups were significantly different (α = 0.05). To evaluate the long-term effect of ultrasound-guided RFA combined with preoperative 3D reconstruction, the prognoses between the RFA and surgery groups were compared. The recurrence and OS rates were calculated by the Kaplan-Meier method. The survival curves were plotted by Kaplan-Meier analysis and compared by the log-rank test. Statistical tests were two-sided and a probability (*p*) value of < 0.05 was considered statistically significant. All analyses were performed using SPSS software version 16.0 (SPSS, Inc., Chicago, IL, USA).

## Results

Concerning the baseline characteristics of two groups, no significant difference was found with respect to age, sex, BMI, fasting blood glucose, HBsAg status, Child-Pugh score, ICG15, and AFP. Surgical duration was remarkably longer in the surgery group versus the RFA group. The postoperative target area was a low-density shadow without enhancement. The complete ablation rate was 94.4% (51/53). In the RFA group, ablation was incomplete in three cases. Artificial hydrothorax was conducted in two cases. Six patients underwent regular hepatic lobectomy and the other 48 underwent non-regular lobectomy. Mean blood loss in the surgery group was about 79.2 mL, with an average Pringle time of 3.8 min (**Table 1**). Also, there was a significant difference in serum levels of TBil (*t* = 2.601, *p* = 0.011), but not WBC, ALT, AST, and Alb, or ΔPT between the two groups before surgery (**Table 2**).

On D1, there were statistically significant differences in all of the above blood sample parameters between the two groups. However, on D3, other than Alb (*t* = 1.114, *p* = 0.268), the serum levels of WBC, ALT, AST, and TBil, and ΔPT were still increased in the surgery group. Furthermore, on D7, WBC, ALT, AST, and ΔPT were still increased in the surgery group, but TBil and Alb between two groups were almost the same (TBil, *t* = 1.420, *p* = 0.158; Alb, *t* = 0.349, *p* = 0.727).

As shown in Table 3, the prevalence of excessive pain, total complications, and total infections was significantly lower in the RFA group as compared to the surgery group (*p* < 0.001). A significant decrease in the duration of the hospital stay after treatment was also observed in the RFA group (*t* = 10.017, *p* < 0.001). The trend seemed to favor a reduction in postoperative mortality in the RFA group (**Table 3**).

**Figure 2:**
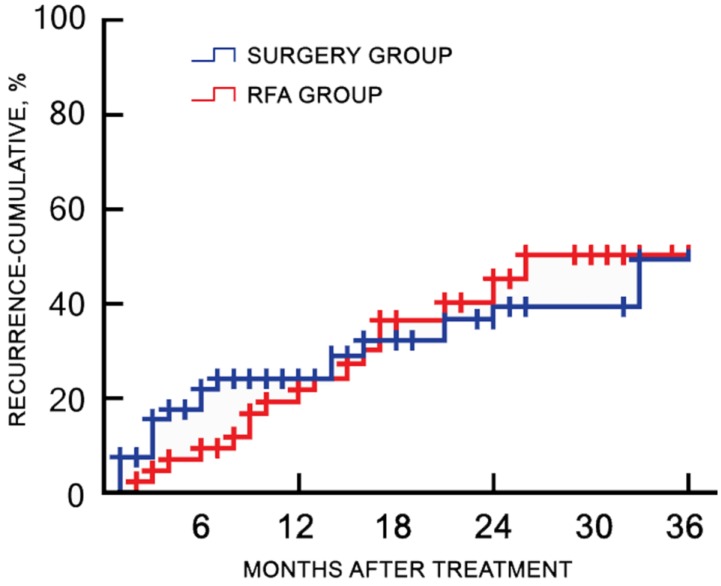
** Cumulative recurrence rates of the RFA and surgery groups after treatment.** There were no significant differences in the cumulative recurrence rate between the two groups during the follow-up period. RFA, radiofrequency ablation.

**Figure 3:**
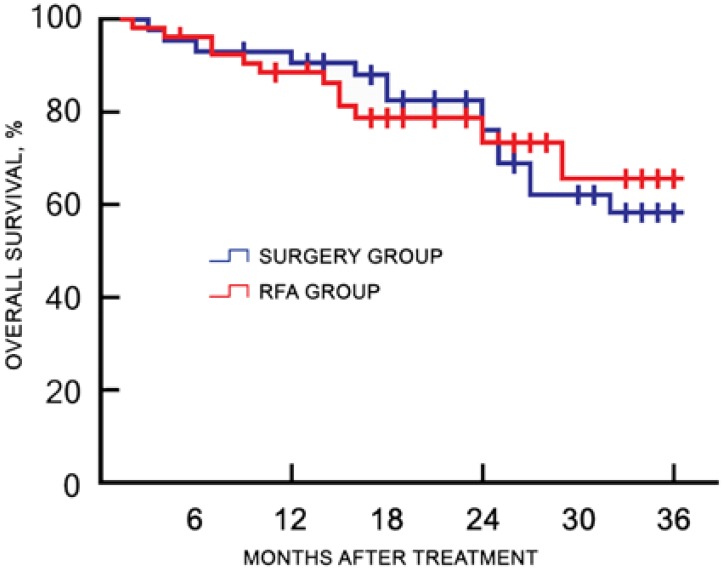
** Kaplan-Meier plot for OS between the RFA and surgery groups after treatment.** OS, overall survival; RFA, radiofrequency ablation.

Of the 120 study participants, 116 (96.7%) were followed-up by phone, and one case in the RFA group and three cases in the surgery group were lost to follow-up. During a median follow-up duration of 22.4 (range, 7.3-45.1) months, no tumor seeding was observed in the RFA group. The 1-, 2-, and 3-year recurrence rates were 18.9%, 45.3%, and 50.9% for the RFA group, and 23.8%, 39.7%, and 49.2% for the surgery group, respectively (*χ2* = 0.217, *p* = 0.641). The corresponding OS rates were 88.7%, 73.6%, and 66.0% for the RFA group, and 93.7%, 76.2%, and 58.7% for the surgery group, respectively (*χ2* = 0.484, *p* = 0.486). There was no significant difference in the cumulative recurrence rates between the two groups over the follow-up period (**Figure 2**). There was no significant difference in the OS rate between two groups, as determined with the log-rank test (**Figure 3**).

## Discussion

In recent years, ultrasound-guided RFA has become an acceptable and frequently used therapeutic option for patients with small HCC. However, for patients with solitary HCC of 3-5 cm who are candidates for both RFA and curative surgical treatment, safety and effectiveness remain controversial. A recent randomized clinical trial conducted in Hong Kong showed that for HCC smaller than 5 cm, or less than 3 tumors, each smaller than 3 cm, the overall tumor recurrence rate was similar in the RFA and resection groups. The 1-, 3-, 5-, and 10-year OS rates were 95.4%, 82.3%, 66.4%, and 41.8 % in the RFA group, compared with 94.5%, 80.6%, 66.5%, and 47.6%, respectively, in the resection group (*p* = 0.531), indicating that long-term survival (10-year OS) was superior in the resection group 10. A large retrospective study, for which patients were divided into groups based on tumor size (i.e., ≤ 20, 21-30, and 31-50 mm), reported that there was no significant difference in OS between the HCC ≤ 20 mm vs. 21-30 mm groups. However, for patients with tumors measuring 31-50 mm, OS was poorer in the RFA group than in the resection group. Interestingly, the worse OS noted with RFA was observed with only a 5-mm increase in tumors measuring > 30 mm 5.

In light of this controversy, we agree that it is crucial to arrive at a comprehensive RFA plan to ensure a tumor-free margin and improve the complete ablation rate. A prospective randomized trial comparing the 3-year clinical outcomes of RFA targeting 5- or 10-mm margins demonstrated that targeting a 10-mm margin could significantly reduce the risk of tumor recurrence 6. Similarly, multivariate analysis from previous studies also revealed that an insufficient margin (5 mm 11 or 3 mm 12) was an independent predisposing factor for overall recurrence after RFA. Our previous study investigating the feasibility of 3D-CT image fusion to assess the ablation margin also demonstrated that a sufficient ablation margin wider than 5 mm was an independent prognostic factor for both OS and PFS 9. Based on the 3D-CT image fusion technique, the current study compared the outcomes of a new method, ultrasound-guided RFA combined with preoperative 3D reconstruction, with surgical resection among patients with solitary HCC of 3-5 cm. The short-term results showed that the incidences of excessive pain, total complications, and total infections were significantly lower in the RFA group than in the surgery group. A significant decrease in the duration of the hospital stay after treatment was also observed in the RFA group. The long-term results with a 3-year follow-up period showed that there were no significant differences in the cumulative recurrence and OS rates between two groups. This result indicated that ultrasound-guided RFA combined with preoperative 3D reconstruction might be a safe and effective therapeutic option for patients with solitary HCC of 3-5 cm.

Conventional ultrasound-guided RFA can only be used to access the ablation margin on two-dimensional images, which may contribute to incomplete ablation particularly for HCC larger than 3 cm. Also, incomplete ablation and sublethal heat treatment could skew HCC cells toward epithelial-mesenchymal transition (EMT) and transform them to a progenitor-like, highly proliferative cellular phenotype both *in vitro* and *in vivo*, which is driven significantly by p46Shc-Erk1/2 13. In addition, incomplete ablation enhanced the invasive and metastatic potential of residual cancer, accompanied with EMT-like phenotype changes by activating β-catenin signaling in HCCLM3 cells 14. Sorafenib reportedly can inhibit the EMT of HepG2 and SMMC7721 cells after insufficient RFA, and might be useful to prevent the progression of HCC after RFA 15. In the present study, ultrasound-guided RFA combined with preoperative 3D reconstruction provided intuitional images of the tumor location, tumor size, and adjacent vasculature in all directions to obtain a comprehensive plan. Afterward, precision and the complete ablation rate can be improved by inserting the electrode needle, while avoiding repeating punctures. Another improved technique, real-time ultrasonography (US)/CT-MRI image fusion-guided RFA, has become increasingly accepted worldwide. A pilot study showed that for HCC smaller than 5 cm, US/CT-MRI image fusion improved tumor visibility and the technical feasibility of RFA. Fusion imaging-guided RFA using multiple electrodes demonstrated a highly effective ablation rate and a low local tumor progression rate during a 2-year follow-up period 16-17.

In the current study, the rate of excessive pain in the RFA group was notably lower than in the surgery group, and the incidence of postoperative complications in RFA group was about 3.7%. No serious complications, such as liver failure, was observed. Surgical experience is an important factor that influences the surgical outcome, but it is immeasurable in ways as clear as, for example, complications are measured in patients 18. The learning curve of hepatectomy is dramatically longer than that of ultrasound-guided RFA. The preoperative 3D reconstruction can help to shorten the learning curve of both RFA and hepatectomy, thereby avoiding artificial influences and experiential differences between different surgeons. Hence, further investigations with larger sample sizes and well-controlled for confounding factors are needed to confirm the long-term effect of ultrasound-guided RFA combined with preoperative 3D reconstruction for solitary HCC of 3-5 cm, so that more surgeons can choose this technique as a first-line or alternative treatment for solitary HCC of 3-5 cm.

## Conclusion

As compared with surgical resection for solitary HCC of 3-5 cm, ultrasound-guided RFA combined with preoperative 3D reconstruction could significantly reduce the morbidity of excessive pain, total complications, and infections, as well as shorten the duration of the hospital stay after treatment. No significant differences were observed in the cumulative recurrence and OS rates during a 3-year follow-up period. Ultrasound-guided RFA combined with preoperative 3D reconstruction appears to be a safe and effective therapeutic option for patients with solitary HCC of 3-5 cm.

## Figures and Tables

**Table 1:** Baseline characteristics of the two groups.

	RFA Group (%) (*n* = 54)	Surgery Group (%) (*n* = 66)	*t/*χ*^2^*	*p*
Age (years)				
≤ 50	46 (85.2)	58 (87.9)	0.186	0.666
> 50	8 (14.8)	8 (12.1)		
Sex				
Male	38 (70.4)	49 (74.2)	0.223	0.637
Female	16 (29.6)	17 (25.8)		
BMI				
≤18.5	8 (14.8)	9 (13.6)	0.486	0.922
18.5-22.9	23 (42.6)	25 (37.9)		
23.0-24.9	20 (37.0)	27 (40.9)		
>25	3 (5.6)	5 (7.6)		
FBG (mmol/L)	4.9 ± 1.9	5.1 ± 1.8	0.591	0.556
HBsAg positive	47 (87.0)	55 (83.3)	0.320	0.572
Child-Pugh score				
Score A	48 (88.9)	61 (92.4)	0.446	0.504
Score B	6 (11.1)	5 (7.6)		
ICG R15 (%)	7.54 ± 4.83	6.37 ± 3.81	1.484	0.141
AFP (ng/mL)				
≤ 20	38 (70.4)	46 (69.7)	0.006	0.936
> 20	16 (29.6)	20 (30.3)		
Surgical duration (min)	72.9 ± 36.3	156.3 ± 73.1	7.644	< 0.001
Complete ablation	51 (94.4)		NA	NA
Blood loss (mL)		79.2 ± 39.2	NA	NA
Pringle time (min)		3.8 ± 1.4	NA	NA

AFP, alpha-fetoprotein; BMI, body mass index; FBG, fasting blood glucose; HBsAg, Hepatitis B surface antigen; ICG R15, indocyanine green retention at 15 min; NA, not available; RFA, radiofrequency ablation.

**Table 2:** Blood sample tests of the two groups before and after surgery.

	RFA Group (%) (*n*=54)	Surgery Group (%) (*n*=66)	*t*	*p*
**WBC (×10^9^/L)**				
Preoperation	6.23 ± 2.89	6.67 ± 3.01	0.811	0.419
D1	13.25 ± 5.39	18.23 ± 7.26	4.184	<0.001
D3	11.75 ± 5.02	14.37 ± 6.19	2.508	0.014
D7	7.26 ± 3.68	9.56 ± 4.69	2.938	0.004
**ALT (IU/L)**				
Preoperation	32.31 ± 14.25	28.19 ± 12.52	1.685	0.095
D1	274.49 ± 129.53	426.53 ± 224.57	4.409	<0.001
D3	126.32 ± 61.12	264.62 ± 127.98	7.286	<0.001
D7	48.91± 21.38	69.91 ± 26.75	4.674	<0.001
**AST (IU/L)**				
Preoperation	34.75 ± 16.83	35.92 ± 17.29	0.373	0.710
D1	247.74 ± 138.03	397.75 ± 213.75	4.452	<0.001
D3	108.96 ± 53.03	227.79 ± 113.84	7.065	<0.001
D7	41.23 ± 23.68	56.68 ± 28.75	3.166	0.002
**TBil (umol/L)**				
Preoperation	17.33 ± 6.76	14.48 ± 5.24	2.601	0.011
D1	26.37 ± 10.65	33.47 ± 17.95	2.560	0.012
D3	18.68 ± 8.53	22.38 ± 10.25	2.119	0.036
D7	16.47 ± 6.83	14.57 ± 7.65	1.420	0.158
**Alb (g/L)**				
Preoperation	35.35 ± 4.54	37.39 ± 6.47	1.956	0.053
D1	32.75 ± 5.47	29.97 ± 4.90	2.934	0.004
D3	33.49 ± 5.87	32.31± 5.69	1.114	0.268
D7	35.76 ± 6.67	36.23 ± 7.83	0.349	0.727
**ΔPT (s)**				
D1	0.73 ± 0.43	0.54 ± 0.33	2.737	0.007
D3	0.32± 0.25	0.15 ± 0.32	3.188	0.002
D7	-0.26 ±0.19	-0.11± 0.21	4.062	<0.001

ΔPT, prothrombin time, true value - upper limit of normal value; Alb, albumin; ALT, alanine aminotransferase; AST, aspartate transaminase; D, day; RFA, radiofrequency ablation; TBil, total bilirubin; WBC, white blood cell count.

**Table 3:** Complications, mortality, and duration of hospital stay after surgery of the two groups

	RFA Group (%) (*n* = 54)	Surgery Group (%) (*n* = 66)
Excessive pain	13 (24.1)	66 (100)
Total complications	2 (3.7)	18 (27.3)
Abdominal hemorrhage	0	2
Upper gastrointestinal hemorrhage	0	1
Biliary leakage	0	1
Liver failure	0	2
Total infection	2 (3.7)	12 (18.2)
Wound infection	0	0
Pulmonary infection	2	11
Abdominal infection	0	1
Sepsis	0	1
Mortality	0 (0)	1 (1.5)
Duration of hospital stay after surgery	2.65 ± 1.79	9.43 ± 4.70

RFA, radiofrequency ablation
